# Scientific Items

**Published:** 1880-07-20

**Authors:** 


### SCIENTIFIC ITEMS.

**Wormlike Derivatives from Blood Corpuscles.**—This very curious observation has recently been made by Dr. Gaule. To what this discovery may ultimately lead, it is impossible to say. Dr. Gaule gives the following directions in order to observe the phenomenon, which are easily fulfilled :

Since animals in captivity do not answer, decapitate a freshly caught, lively frog, and catch the blood in a glass stoppered bottle containing some mercury and about 5 cubic centimeters of a solution of salt (6 per cent.) The blood is now defibrinated by shaking it with the mercury, and hereupon a drop is placed upon the warm stage, the thermometer of which shows 30° to 32° C. These conditions may be varied to some extent, though with more risk of failure.

On watching this blood while warm, there appears in a blood corpuscle on the side of the nucleus, a rod-shaped body, containing some striæ, which higher lenses prove to be minute globules. This little rod is about one-half the length of the corpuscle, with tapering ends, its body showing a bluish lustre. It begins to raise itself out of the corpuscle, twists about and finally leaves it.

When once liberated, it looks, in the words of the author, more like a "little worm" than anything else. It now begins to shoot about, rushing towards other corpuscles and apparently playing with them. It is evidently "sticky" since corpuscles and other microscopic objects adhere to it.

After continuing to "play" for some minutes, these little worms finally lose their mobility and distinctness, and ultimately disappear, apparently by dissolving in the serum.

The number of corpuscles giving birth to these worms varies; under favorable circumstances nearly all participate. The corpuscles themselves undergo some changes in shape and appearance and finally disappear like their derivatives.

While admitting the puzzling and as yet inexplicable nature of the phenomenon, Gaule considers the worm "to represent the original protoplasm of the corpuscle." In this connection reference is made to an article by Prof. R. Arndt on the red corpuscles, in which that writer throws out the suggestion that the spirilæ found in the blood of relapsing fever are but derivatives of the red corpuscles. He adduces, however, merely ingenious "circumstantial evidence," but no direct proof.—*Chic. Med. Review.*

**Dynamite for Stumps.**—A correspondent of an English agricultural journal gives the following account of his use of dynamite for removing stumps of trees felled in a park at Mentmore, Bucks, in order to improve the landscape and leave more room for the rest of the trees to develop themselves :

The only tools required are an earth auger, which is similar to an old-fashioned wood auger, 2 inches diameter at the bit end, about 4 feet long, and fitted with a slightly hollowed shield or cap, which the man fits against his chest when boring (this is used for boring holes between the fangs), a crow-bar, a grafting and a stock axe.

Suppose a large root is to be removed out of the ground: a hole is made with the earth auger between two of the strongest fangs; this is put in at an angle, so that the bottom of the hole is as near under the center of the root as is possible. The hole is then charged with a few cartridges of dynamite, according to the size and strength of the root; a primer cartridge, containing cap and fuse, is then inserted on the top of the charge, and the whole rammed down with loose earth by a wooden rammer. The end of the fuse is then lighted ; this explodes the cap, and that in its turn the dynamite, and the whole mass is usually blown out, breaking the root into convenient pieces for loading up or burning. The fuse is cut off at convenient length so as to allow the workmen to get out of danger, which is usually from 50 to 100 yards, according to the strength of the charge.

After the charge has exploded, seldom anything remains but a large hole, much resembling the bed of a boiler. I took particular notice that no damage whatever was done to the surrounding trees. We had nearly 400 roots got out by this process, and with two of our common laboring men, with one man sent by the agents of the Dynamite Company, we have been able to remove from twenty-five to thirty per day of roots averaging from a foot and a half to four feet and a half in diameter. I find from careful calculations made that we have been enabled to remove the roots in a far more expeditious manner than hitherto, and at from 50 to 60 per cent. less cost.

No one need be prevented from using dynamite on the score of its being dangerous, for with ordinary care it is, in my opinion, as safe to use as gunpowder.—*Journal of Chemistry.*

**Light and Heat.**—If a piece of wood be placed in a decanter of water, and the focus of a large burning glass be thrown upon it, the wood will be completely charred, though the sides of the decanter through which the rays pass will not be cracked, nor in any way affected, nor the water perceptibly warmed. If the wood be taken out, and the rays be thrown on the water, neither the vessel nor its contents will be in the least affected; but if a piece of metal be put into the water, it soon becomes too hot to be touched, and the water will presently boil. Though pure water alone, contained in a transparent vessel, cannot be heated, yet, if by a little ink it may be made of a dark color, or the vessel itself be blackened, the effect will speedily take place.— *Young Scientist.*

**Seeing by Telegraph.**—We read in the Times of a novel and startling addition to telegraphic possibilities, viz., seeing by telegraph. By means of a lens, an image of the object is thrown upon a receiving plate. This is built up of a series of thermopile elements grooved anteriorly to an even surface and connected by their posterior ends with a series of wires which transmit the electric currents generated by the reception of the image to a similar series of elements in a second plate at a distance. In this second plate the electric currents create changes exactly corresponding to those produced by the image in the receiving plate.

The close analogy between this apparatus and the rods and cones of the retina and the fibres of the optic nerve is obvious.—*Brit. Medical Journal.*

